# SARS-CoV-2 and Emerging Foodborne Pathogens: Intriguing Commonalities and Obvious Differences

**DOI:** 10.3390/pathogens11080837

**Published:** 2022-07-27

**Authors:** Ahmed G. Abdelhamid, Julia N. Faraone, John P. Evans, Shan-Lu Liu, Ahmed E. Yousef

**Affiliations:** 1 Department of Food Science and Technology, The Ohio State University, Columbus, OH 43210, USA; abdelhamid.9@osu.edu; 2 Botany and Microbiology Department, Faculty of Science, Benha University, Benha 13518, Egypt; 3 Molecular, Cellular and Developmental Biology Program, The Ohio State University, Columbus, OH 43210, USA; faraone.7@buckeyemail.osu.edu (J.N.F.); evans.2381@buckeyemail.osu.edu (J.P.E.); 4 Center for Retrovirus Research, The Ohio State University, Columbus, OH 43210, USA; liu.6244@osu.edu; 5 Department of Veterinary Biosciences, College of Veterinary Medicine, The Ohio State University, Columbus, OH 43210, USA; 6 Viruses and Emerging Pathogens Program, Infectious Diseases Institute, The Ohio State University, Columbus, OH 43210, USA; 7 Department of Microbial Infection and Immunity, The Ohio State University, Columbus, OH 43210, USA; 8 Department of Microbiology, The Ohio State University, Columbus, OH 43210, USA

**Keywords:** SARS-CoV-2, Shiga toxin-producing *Escherichia coli*, Shiga toxin, COVID-19, infectious diseases

## Abstract

The coronavirus disease 2019 (COVID-19) has resulted in tremendous human and economic losses around the globe. The pandemic is caused by the severe acute respiratory syndrome coronavirus 2 (SARS-CoV-2), a virus that is closely related to SARS-CoV and other human and animal coronaviruses. Although foodborne diseases are rarely of pandemic proportions, some of the causative agents emerge in a manner remarkably similar to what was observed recently with SARS-CoV-2. For example, Shiga toxin-producing *Escherichia coli* (STEC), the most common cause of hemolytic uremic syndrome, shares evolution, pathogenesis, and immune evasion similarities with SARS-CoV-2. Both agents evolved over time in animal hosts, and during infection, they bind to specific receptors on the host cell’s membrane and develop host adaptation mechanisms. Mechanisms such as point mutations and gene loss/genetic acquisition are the main driving forces for the evolution of SARS-CoV-2 and STEC. Both pathogens affect multiple body organs, and the resulting diseases are not completely cured with non-vaccine therapeutics. However, SARS-CoV-2 and STEC obviously differ in the nature of the infectious agent (i.e., virus vs. bacterium), disease epidemiological details (e.g., transmission vehicle and symptoms onset time), and disease severity. SARS-CoV-2 triggered a global pandemic while STEC led to limited, but sometimes serious, disease outbreaks. The current review compares several key aspects of these two pathogenic agents, including the underlying mechanisms of emergence, the driving forces for evolution, pathogenic mechanisms, and the host immune responses. We ask what can be learned from the emergence of both infectious agents in order to alleviate future outbreaks or pandemics.

## 1. Introduction

In early 2020, the entire globe experienced unforeseen lockdown and exceptional loss of human lives. This crisis was triggered by the emergence of an infectious agent, the severe acute respiratory syndrome coronavirus 2 (SARS-CoV-2), which caused the COVID-19 pandemic. The heavy toll associated with the emergence of such disease stems from the lack of prior body immunity, predictable preventive measures, and readiness of medical treatments. SARS-CoV-2 is a single-stranded positive-sense RNA-enveloped virus that belongs to the family *Coronaviridae* and genus *Betacoronavirus* [1,2]. Members of this genus are subject to a relatively high incidence of mutation and recombination events, resulting in increased capability of transmission in human populations [3]. COVID-19 is a disease with a wide range of symptomatology, but it can also be asymptomatic. When symptomatic, the infected individual experiences some combination of fever/chills, cough, shortness of breath, muscle and body aches, loss of taste or smell, nausea or vomiting, and diarrhea. These symptoms can lead to respiratory impairment or failure, acute respiratory distress syndrome, septic shock or multiple organ dysfunction, and death. Persons at high risk of complication are the elderly (>65 years) or those with comorbidities such as immunodeficiency, cardiovascular disease, lung disease, pregnancy, obesity, or being a smoker [4].

Throughout history, humans have confronted many emerging infectious diseases (EID) with similarities to COVID-19. In this review, EID caused by pathogens that emerged from an animal host and used humans as an incidental host will be emphasized. Some foodborne EIDs are recognized to have zoonotic potential, which largely impacts their ability to cause diseases and facilitates their evolutionary shifts. Many emerging foodborne pathogens are unavoidable, widely spread, and constitute significant public health risk. Each year, it is estimated that foodborne pathogens cause 600 million illnesses and 420,000 deaths globally [5]. Although considerable progress has been made to mitigate these diseases, the evolutionary race between causative pathogens and their hosts is ongoing and the outcome of this race is always unpredictable. Notably, some foodborne pathogens, particularly the enterohemorrhagic *Escherichia coli*, and SARS-CoV-2 have emerged through similar evolutionary mechanisms using multiple hosts, targeted body organs beyond their initial entry sites, and similarly evoked certain host responses. This review aims to compare these seemingly different pathogens by emphasizing their commonalities so that the preparedness for future disease pandemics or outbreaks can be improved.

## 2. Zoonotic Transmission

SARS-CoV-2 and some foodborne pathogens have emerged as zoonotic infectious agents, i.e., they are transmissible from animals to humans. SARS-CoV-2 is the latest example of a coronavirus that has a zoonotic origin in bats. In 2002–2003, SARS-CoV emerged, likely from bats and used civets as the intermediate host [6], and caused a large respiratory disease outbreak in Asia. Similarly, the Middle East respiratory syndrome coronavirus (MERS-CoV) emerged in 2012, where the camel was the intermediate host [7]. Other viruses such as human immunodeficiency virus-1 (HIV-1) emerged at least 4 times early in the 20th century, likely through bush meat hunting in Africa, whereas HIV-2 emerged at least 8 times [8]. Ebola viruses have had multiple spillover events in Africa since the 1970s; however, it is unclear what the sources of these spillover events are, as most animals identified as being infected are too symptomatic to effectively spread the virus [9]. The 1918 influenza pandemic resulted in nearly 50 million deaths worldwide, and is thought to have originated from swine [10]. In a similar fashion, avian influenza A viruses have emerged multiple times since 1997, and the source was presumed to be wild aquatic birds [11].

New infectious agents associated with foodborne diseases have emerged in the past four decades. *E. coli* O157:H7, *Cyclospora cayetanensis*, *Vibrio vulnificus*, and *Salmonella enterica* serovar Enteritidis are examples of these emerging foodborne pathogens [12]. A common characteristic among these diverse pathogens is that they have animal reservoirs. The pathogenicity of *E. coli* O157:H7, a Shiga toxin-producing bacterium, was revealed in the early 1980s when the microorganism was associated with an outbreak of bloody diarrhea due to consumption of hamburgers [13]. The bacterium was found to have come from cattle [14]. *Cyclospora* spp. emerged as a new pathogen when it was implicated in a 1992 disease outbreak due to consumption of contaminated raspberries; it was suggested that the pathogen has an avian reservoir [15,16]. *Vibrio vulnificus* was recognized in patients who had eaten raw oysters and the pathogen was thought to have a reservoir in shellfish [17]. *Salmonella* Enteritidis was repeatedly isolated in the New England region in 1978 before a salmonellosis outbreak appeared four years later and was traced back to eggs [12]. Afterwards, *Salmonella* Enteritidis was documented to be prevalent in poultry [18,19] and the pathogen has become dominant in the United States since then.

A representative example of foodborne zoonotic pathogens with an intriguing history of evolution is the Shiga toxin-producing *E. coli* (STEC). Formerly known as enterohemorrhagic *E. coli* (EHEC), STEC is one of several gastrointestinal pathogenic *E. coli* groups (pathotypes), which also include enteropathogenic, enterotoxigenic, enteroinvasive, and enteroaggregative *E. coli* [20]. Strains of *E. coli* were traditionally grouped into serotypes based on the somatic (O) and the flagellar (H) antigen that each strain carries, and multiple serotypes may belong to a given pathotype. A famous STEC serotype is *E. coli* O157:H7, and the first strain to be recognized was *E. coli* O157:H7 EDL-933.

The STEC pathotype has evolved in a manner reminiscent of the events that led to the emergence of SARS-CoV-2. New STEC clones are continuously identified while emerging SARS-CoV-2 variants are constantly being isolated. As domestication of animals increases, host switching by such pathogens becomes more feasible. Comparing and contrasting STEC and SARS-CoV-2 (Table 1) may help in alleviating future devastating disease outbreaks.

## 3. Evolution of the Two Pathogens Compared

Since the first occurrence of COVID-19 and STEC-related disease outbreaks, new variants of SARS-CoV-2 and strains of STEC were reported in rapid succession. There is no doubt that some of these variants and strains were in existence before the associated diseases were first reported. Because new variants and strains are expected to be revealed in the future, it is of value to shed some light on those that evolved or were discovered since the advent of COVID-19 and STEC-related illnesses.

After the discovery of STEC O157:H7 in 1982, other similarly pathogenic STEC serotypes have been revealed. One of the greatest public health concerns is the serotype *E. coli* O26:H11 and its nonmotile counterpart (*E. coli* O26:H^−^); these emerged after 1990 as the most common non-O157 STEC serotypes in Europe and the United States [43]. The STEC O26 strains were associated with hemolytic uremic syndrome (HUS) with simultaneous occurrence of hemolytic anemia, thrombocytopenia, and renal failure [44]. Genomes of STEC O26 strains contained prophages and plasmids, and the diversity of this mobilome accounts for the overall intra-serotype diversity [45].

In addition to STEC O157 and O26, other serogroups emerged, and these include *E. coli* O145, O103, O111, O121, O91, and O45 [46,47]. A devastating STEC-related disease outbreak occurred in 2011 in Germany and caused 4000 illnesses and more than 50 deaths. This disease outbreak was attributed to an emerging STEC serotype, namely *E. coli* O104:H4, which was associated with high morbidity and mortality, and exceptional virulence characteristics [48]. It was proposed that the virulence characteristics known of EHEC, enteroaggregative *E. coli* (EAEC), and extra-intestinal pathogenic *E. coli* were combined into *E. coli* O104:H4, which is capable of Stx2a production and EAEC-like adherence, and has the iron-capturing system, aerobactin, and yersiniabactin [45]. The combination of these factors is what makes this serotype highly virulent.

Recently, a new STEC hybrid evolved and demonstrated the ability to cause severe HUS and invasive infections. This hybrid is *E. coli* O80:H2, which appeared in several European countries and was found to harbor virulence factors such as Stx, enterohemolysin, intimin, and a large plasmid (>100 kb). The plasmid also contains several virulence factors such as serum resistance protein, a hemolysin, a putative secretion system, salmochelin, and aerobactin. Additionally, the plasmid harbors genetic traits conferring resistance to several drugs such as streptomycin, tetracyclines, penicillins, and cotrimoxazole [45,49]. STEC O80:H2 seems to have high genetic flexibility since it can integrate various antibiotic resistance mechanisms [50]. Thus, the evolution of STEC via crossing of the boundaries between different *E. coli* pathotypes and serogroups is alarming; this evolution may lead to the emergence of new virulent clones with intricate virulence mechanisms.

The considerable ability of STEC to develop highly virulent clones seems to parallel that of SARS-CoV-2. The large number of sequenced SARS-CoV-2 genomes provided evidence of continuous development of variants; some of these differ only in specific genomic sites by single nucleotide polymorphisms (SNPs) [51]. A variant of SARS-CoV-2 is defined as a virus genome that contains one or more mutations [52]. These variants demonstrated increased transmissibility, greater disease severity (e.g., increased hospitalizations or deaths), a significant reduction in neutralization by antibodies generated during previous infection or vaccination, reduced effectiveness of treatments or vaccines, or diagnostic detection failures [52]. The S protein has developed multiple SNPs that could impact the ability of the virus to enter human cells. Several variants of concern resulted from changes in specific residues in the S protein. For example, SNP at residue 614 occurred when the site changed from aspartate [D] to a glycine [G]; thus, this SNP is referred to as “D614G” [51,53]. Additional variants were observed due to SNPs in the S protein such as N501Y, E484K, E484Q, T478K, K417T, K417N, S477N, and L452R, among others [1,51].

The evolution of STEC and SARS-CoV-2 is triggered by several genetic mechanisms, including the acquisition of new genes, gene deletion resulting in loss of biological function, and point mutations. Considerable parallels and profound differences in these mechanisms are discussed below in relevance to the evolution of STEC and SARS-CoV-2.

### 3.1. Gene Acquisition

**Figure 1:**
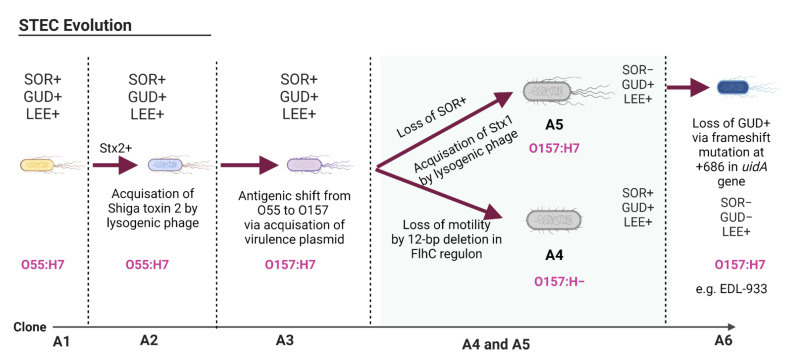
Evolution of Shiga toxin-producing *Escherichia coli* (STEC) O157:H7. A1–A6 represent clonal complexes originating from the ancestral clone O55:H7 to the typical Shiga toxin-producing *E. coli* O157:H7. Abbreviations: SOR, sorbitol fermenting ability; GUD, glucuronidase activity; LEE, locus of enterocyte effacement; Stx1, Shiga toxin 1; Stx2, Shiga toxin 2. The figure was adapted from [58] and created using Biorender.com.

Gene acquisition is an important force driving the evolution of pathogens, including STEC and SARS-CoV-2. Both pathogens acquired genes from non-distant clones and this acquisition improved their fitness to host targets. However, there are obvious differences in the gene acquisition routes and speed in these two pathogens. STEC has a genome size of 5.5–5.9 Mb, which is much larger than the genome of other *E. coli* strains such as *E. coli* k12 [54]. The STEC genome shares nearly 4.1 Mb as the conserved backbone sequence with most *E. coli* strains, while the remainder of the genome (≥1.4 Mb) is an acquired foreign DNA [55]. STEC evolution was prompted by the acquisition of new genes via genetic transfer by mobile elements such as phages, plasmids, or integrative elements [38,39]. A single or a combination of these genetic transfer events may have been the driving force of the emergence of new STEC serotypes. The evolution of STEC O157:H7 was postulated to occur through a complex cascade of events (Figure 1). It is believed that *E. coli* O157:H7 originated from the ancestral clonal complex, *E. coli* O55:H7 (designated as A1 clone), which possesses a sorbitol-fermenting ability (SOR+) and glucuronidase activity (GUD+), in addition to the locus of enterocyte effacement (LEE+) genomic site [56,57]. It was hypothesized that the A2 (SOR+, GUD+, LEE+) clonal complex was derivatized from A1 via the acquisition of the Shiga toxin 2 gene (*stx2*) by a transduction mechanism resulting from infection by a lysogenic phage [58]. The A3 clone (SOR+, GUD+, LEE+) evolved from A2 through the acquisition of a virulence plasmid (pO157), which led to an antigenic shift from serotype O55:H7 to serotype O157:H7 [58,59]. The virulence plasmid pO157 comprises 100 open reading frames (ORFs) that are thought to originate from a different species. Some of these ORFs (~35) are involved in the pathogenicity of STEC [55]. Two unique lineages were diverted from the A3 clone: one is flagellated and the other is not (Figure 1); these evolved thorough gene deletions or point mutations as described in the latter section.

**Figure 2:**
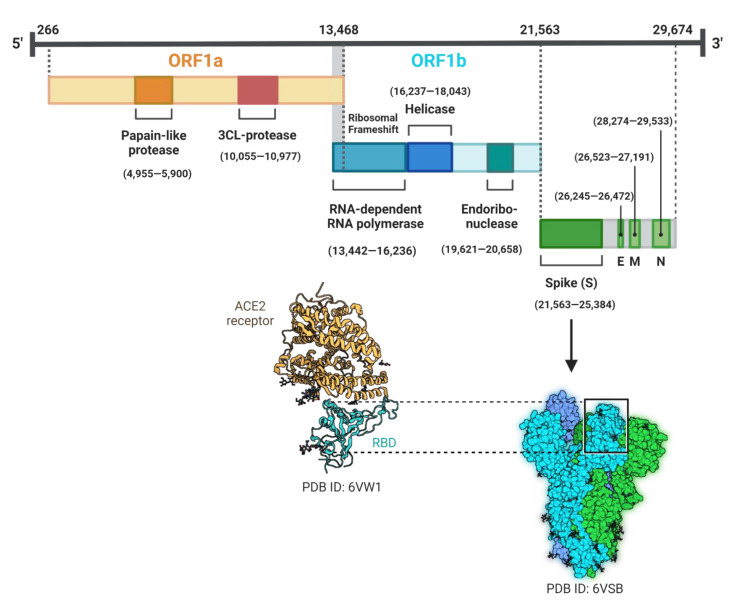
SARS-CoV-2 genome, which shows single-stranded RNA that encodes open reading frames (ORFs) 1a and 1b, spike (S), envelope (E), membrane (M), and nucleocapsid (N) proteins. Three-dimensional structure (protein database ID number 6VW1) for the receptor-binding domain (RBD) interacting with the human angiotensin converting enzyme (ACE2) receptor is illustrated. The figure is adapted from [63] and created using Biorender.com.

Although the SARS-CoV-2 genome is much smaller than that of STEC, and the virus genome (~30 kb) consists of six ORFs only, it is the largest among RNA viruses. The genome encodes several structural and non-structural proteins, among which spike (S), membrane (M), envelope (E), and nucleocapsid (N) proteins (Figure 2) are the structural proteins [60,61,62,63]. The phylogenetic comparisons of the virus and other coronaviruses revealed gene acquisition mechanisms critical to viral evolution, namely genomic recombination events [40,41]. The highest frequency of recombination events occurred in the ORF1b and then the N-terminus of the S protein [40]. Recombination in the S protein is of critical importance for adaptation following a zoonotic transmission since the protein is essential for virus entry via binding to host Angiotensin-converting enzyme 2 (ACE2) receptor with altered affinity [2,23,64]. Despite its overall sequence similarity to the bat coronavirus, RaTG13, the receptor-binding domain (RBD) of the S protein in SARS-CoV-2 was more divergent from that of RaTG13. It appears that the S protein region consists of several segments that have different phylogenetic relations when different *Sarbevirus* strains were compared [40]. Four genomic regions in SARS-CoV-2 have undergone recombination, with three of these regions showing similarity to those in bat coronavirus RaTG13 [40]. One 222-nucleotide region in the S protein gene encodes several amino acid residues of the RBD and showed closest similarity to that of the pangolin Guangdong 2019 strain [40,65]. This finding suggests that the RBD region has a unique evolutionary history compared to the rest of the S protein, implying that recombination between pangolin-CoV and a SARS-CoV-2 ancestral strain could have occurred inside the bat or other intermediate host [1]. Additionally, phylogenic analyses of the whole S region indicate that genetic recombination between coronaviruses from distantly related mammals likely has occurred. This scenario is important particularly, as it informs how working closely with wild animals could have contributed to SARS-CoV-2 evolution.

Recombination also contributes to the diversity of the S1/S2 cleavage site in coronaviruses, and this site is also subject to other genetic modifications such as substitutions and deletions [66]. The S1/S2 cleavage site is evolutionarily important in coronaviruses [66]. A multiple sequence alignment of the S protein S1/S2 region among several coronaviruses revealed that SARS-CoV-2 is closely-related to SARS-CoV and the bat coronavirus RaTG13 [67]. The S1/S2 site is cleaved by furin, a human protease, and other host proteases to release the S2 fusion peptide, which is required for fusion with the host cell membrane and virus entry. Intriguingly, recombination between the Delta and Omicron SARS-CoV-2 variants has created a hybrid spike protein in the new SARS-CoV-2 Delta-Omicron variant (known as Deltacron) in the United States and other countries [68]. Additionally, two Deltacron recombinant viruses, in which the 5′-end of the viral genome was acquired from the Delta genome and the 3′-end was from Omicron, have been reported in the US [69]. In another study, researchers identified several amino acid mutations characteristic of Delta S protein in the Omicron isolates [70]. Interestingly, recent reports described recombination between Omicron sublineages (e.g., BA.1 and BA.2), where BA.1 acts as the acceptor. For example, BA.1/BA.2 recombinant was detected in Hong Kong, and the breakpoint was near the 5′ end of the S gene [71]. These pieces of evidence imply that recombination between SARS-CoV-2 variants results in new variants with altered pathogenicity and transmission attributes [72]. Thus, recombination is deemed as a main evolutionary tool for SARS-CoV-2 variants.

### 3.2. Gene Deletion and Loss of Function

Considering the large differences in genome sizes, gene deletion was a more significant evolutionary driving force in STEC than in SARS-CoV-2. Admittedly, some of the deletions in the STEC genome reshaped the pathogen’s phenotypic traits that are not crucial to its virulence. STEC evolution occurred via the inactivation of genes whose functions are not compatible with virulence expression [73]. As shown in Figure 1, two lineages were derivatized from the A3 clonal complex. The first lineage is the A4 clone (SOR+, GUD+, LEE+), which evolved from A3 by loss of motility because of a 12-bp deletion in the *flhC* regulon gene [74]. The clonal complex A4, also known as SFO157, included strains such as 493–89 or H2687; these were isolated in Germany and Scotland in 1989 and 2003, respectively [57]. The second lineage, A5 (SOR−, GUD+, LEE+), emerged from A3 by acquisition of the Stx1 gene and loss of the sorbitol-fermenting phenotype [58]. The development of the sorbitol-negative phenotype of STEC O157 was thought to be due to the truncation in the *srlA* and *srlE*, which encode the glucitol/sorbitol-specific phosphotransferase system. The role of sorbitol fermentation in STEC pathogenesis is not fully understood since both sorbitol-positive and sorbitol-negative STEC phenotypes were implicated in human illnesses. Schutz et al. [75], however, argued that the loss of the sorbitol-positive phenotype may correlate with the ability of STEC to colonize a new animal host or transmit between animal hosts effectively, but this hypothesis needs further investigation. The loss of gene function contributed to the development of the A6 clone of STEC. This was evident when the A6 clone was derivatized from the A5 clonal complex by loss of glucuronidase activity due to a frameshift mutation at the +686 position in the *uidA* gene, which encodes for beta-glucuronidase [76] as shown in Figure 1. The A6 clonal complex contains the typical STEC O157:H7 strains such as EDL–933 and Sakai, which were identified in the USA and Japan in 1982 and 1996, respectively [57].

Gene deletions or truncations are not likely to be a major evolutionary mechanism in SARS-CoV-2 since most, if not all, genes of the virus are seemingly necessary for persistence and host adaptation. However, it was reported that deletion in the NSP1 region, particularly Δ500–532, was found in clones from several countries; this deletion was associated with a lower viral load in SARS-CoV-2 infections [77]. Similarly, researchers in Bangladesh found that deletions in ORF7, ORF8, and ORF10 were associated with reduced virulence of the virus [78,79]. Other researchers indicated that isolates with deletions in or close to the furin cleavage site were associated with mild or asymptomatic outcomes [80]. Deletions of few amino acid residues in the S gene, particularly the N-terminal region, have been reported in most variants of concern (Alpha, Delta, Omicron BA.1 and BA.2); these deletions can enhance virus neutralization resistance [81] and infectivity [82]. It is generally believed that SARS-CoV-2 variants with gene deletions are less likely to survive or to circulate for a relatively long time during the pandemic, though small deletions can have their benefits.

### 3.3. Point Mutations

Point mutations, which change gene functions [83], were more crucial in the emergence of SARS-CoV-2 than in STEC. Diversifying selection via point mutations is one of the evolutionary forces in SARS-CoV-2. Comparative analyses of SARS-CoV-2 with the closest known coronaviruses emphasize the evolution via diversifying selection and divergence from the closest relative, which is a coronavirus from a bat strain in Yunnan, China [1,84]. The bat coronavirus RaTG13 and its genome shares ~96% sequence similarity to SARS-CoV-2 [60,85]. Despite such overall genomic similarity, the RBD region of the strain RaTG13 has low similarity to that of SARS-CoV-2 and the bat virus RBD has limited affinity to the human ACE2 receptor. In addition, SARS-CoV-2 can poorly infect bats or bat cells. This may imply that some bat viruses with RBD genetically close to that of SARS-CoV-2 are missing in the evolution story of SARS-CoV-2 [86]. Based on prior knowledge of the speed by which coronaviruses accumulate nucleotide substitutions over time, one can determine the time to the most recent common ancestor of SARS-CoV-2 and the RaTG13 strain. One way to look at this is to use synonymous versus nonsynonymous nucleotide substitutions that can occur in the SARS-CoV-2 genome. Synonymous changes are those nucleic acid changes that do not alter the amino acid sequence, whereas nonsynonymous changes do [1]. With the aid of comparative genomic analyses, it is possible to estimate the rate of nonsynonymous substitutions and of synonymous changes and observe the selective pressures exerted [84]. In the SARS-CoV-2 S gene, the ratio of the nonsynonymous to the synonymous substitutions per site increased from 1.01 to 2.46 as the COVID-19 disease progressed over time [87]; this increase signifies that the S gene was under strong positive selection. Another way to estimate the time of divergence of SARS-CoV-2 from its closest relative is to simply use the synonymous substitutions. Utilizing this principle, the divergence time between SARS-CoV-2 and the RaTG13 strain is 51.71 years (28.11–75.31, 95% CI) according to Wang et al. [88]; other researchers reported a range of 18–71 years for this divergence time [1,89].

**Figure 3:**
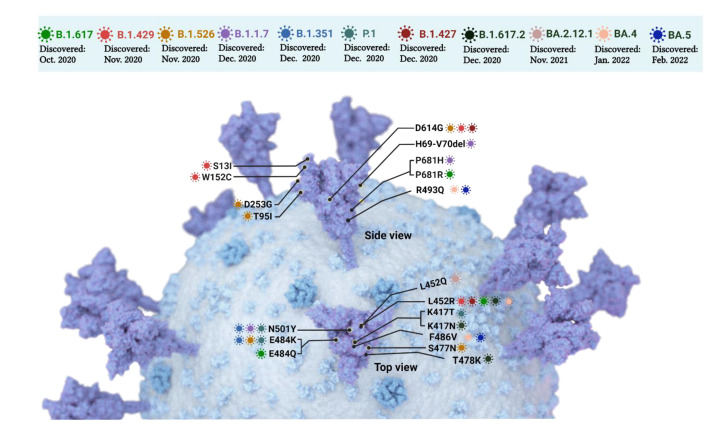
Variants of concern of SARS-CoV-2 as of February 2022. Variants are caused by single nucleotide polymorphisms (SNPs) at particular sites on the spike protein, resulting in changes in the amino acid residues at these sites. For example, a variant where arginine (R) is substituted for leucine (L) at residue 452 is denoted by L452R. Another variant is caused by the deletion of the H69/V70 site. The amino acid residues shown are C, D, E, G, H, I, K, L, N, P, Q, R, S, T, V, W, and Y, which denotes for cysteine, aspartic acid, glutamic acid, glycine, histidine, isoleucine, lysine, leucine, asparagine, proline, glutamine, arginine, serine, threonine, valine, tryptophan, and tyrosine, respectively. Key mutations for each variant are presented and only the most recent omicron variants (BA.2.12.1, BA.4, and BA.5) with highest transmissibility are illustrated. The figure was created using Biorender.com.

Over the course of the COVID-19 pandemic, the SARS-CoV-2 spike gene has accumulated several mutations (Figure 3) that have increased virus transmissibility and/or ability to escape from neutralizing antibodies. Early in the pandemic, the D614G substitution emerged and greatly enhanced viral replication in lung epithelial cells and increased the stability of the virions, virus infectivity, and transmission [90,91]. Since its initial occurrence, this mutation is preserved in nearly every emerging variant. Subsequently, the Alpha (B.1.1.7) variant emerged and was characterized by three additional mutations: a deletion of H69-V70, N501Y, and P681H, which enhanced the virus infectivity and neutralization resistance. These mutations allowed the Alpha variant to spread rapidly across the globe, quickly bringing it to dominance [92]. Soon after, the Beta (B.1.351) variant emerged, demonstrating marked neutralization escape with the addition of the mutations K417N and E484K (Figure 3); however, it appears this adaptation was at the expense of the virus infectivity, preventing Beta from becoming a dominant variant [93].

The emergence of the Delta variant marked the subsequent wave of COVID-19, exhibiting a combination of neutralization resistance and increased transmissibility with key mutations: L452R, T478K, and P681R (Figure 3), which quickly made it the dominant strain worldwide [94]. Finally, the outbreaks of the Omicron subvariants are ongoing. Omicron represents a shift away from the previous variants, being characterized by nearly three times the mutations (Figure 3) in the spike gene. The subvariants include several mutations at residues already characterized in studies of the other variants, including T478K, K417N, E484A, L452Q, F486V, R493Q, N501Y, and P681H [95,96,97]. These mutations, known to provide increased infectivity and decreased neutralization, are paired with a myriad of uncharacterized mutations, endangering the efficacy of current public health efforts against Omicron subvariants, including BA.2.12.1 and BA.4 and BA.5, which recently emerged [98]. Overall, mutations in the S protein result in new molecular characteristics that impact the protein-antibody complex, and hence enhance the variant’s immune escape and may boost the variant transmission, particularly in case of Omicron (R_0_ 10 compared to the original strain of SARS-CoV-2 with R_0_ 2.5) [99,100]. Variants with L452R, T478K, E484K/Q/A, and N501Y SNPs showed reduced neutralization by some monoclonal antibodies and by COVID-19 patient sera and mRNA vaccine recipient sera, and such SARS-CoV-2 variants are circulating [101,102]. Moreover, Delta-Omicron recombinant SARS-CoV-2 displays remarkable resistance to neutralization by sera from individuals who received two doses of mRNA vaccine and sera from COVID-19 patients during the Delta variant wave. However, the breadth of the memory B cell repertoire following the third mRNA vaccine dose could provide sufficient memory for broadly neutralizing antibodies as evident by recent studies [37,103]. Fortunately, sera from the recipients of the third dose showed a less severe drop in neutralizing antibody titers and incomplete antibody escape [104]. Despite this good news, it is plausible to ask if Omicron-based vaccines should be developed, or if there is a demand to switch from antibody-based therapy to treatment with small molecules that target non-structural proteins. The latter strategy is under investigation, and some research indicated strong antiviral activity of the 3CL protease inhibitor, Nirmatrelvir, and the RdRp inhibitor, Molnupiravir, against the Omicron variant [105].

## 4. Pathogenesis and Immune Evasion

**Figure 4:**
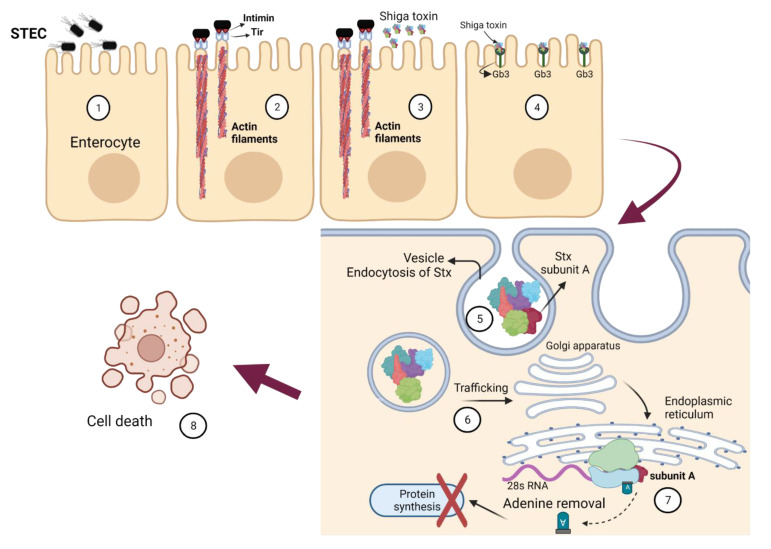
The mode of action of Shiga toxin. (1) Initial contact of Shiga toxin-producing *Escherichia coli* (STEC) with enterocyte. (2) Attachment of STEC to the cell via intimin and Tir protein interaction, and actin polymerization. (3) Production of Shiga toxin. (4) Binding of Shiga toxin to globotriaosylceramide (Gb3) receptor. (5) Endocytosis of Shiga toxin into the enterocyte via vesicle formation. (6) Transport of the Shiga toxin to the Golgi apparatus, then to the endoplasmic reticulum. (7) Release of the Shiga toxin subunit A, which acts as N-glycosidase by removing adenine from the ribosomal 28s RNA; this abolishes translation of the mRNA by the ribosome, halting protein synthesis and leading to cell death (8). The figure was created using Biorender.com.

**Figure 5:**
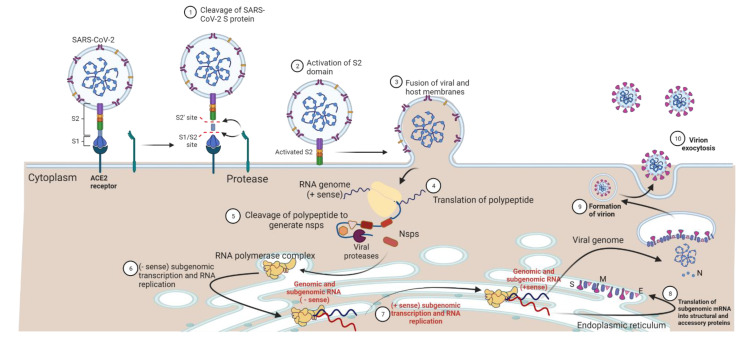
Infection of the mammalian cell by SARS-CoV-2 and virions multiplication. Initially, the virus binds to an angiotensin converting enzyme (ACE2) receptor on the cell surface, followed by processing of the viral S protein by host proteases, leading to the fusion of the viral envelope and host cell membrane. Thereafter, the viral genomic RNA is deposited into the host cell cytoplasm and translated by the host translation system. The resulting viral polypeptide is cleaved by proteases encoded on the polypeptide itself, and the cleaved components generate RNA polymerase complex. The latter uses the virus RNA genome (+ sense) as a template to generate negative-sense RNA genomes and subgenomic RNA regions before both types of RNA serve as templates for synthesis of positive-sense full-length RNA genome and subgenomic mRNAs. These mRNAs act as templates for the synthesis of structural and accessory proteins, which decorate the viral nucleocapsid. The whole transcription and translation process of the viral subgenomic RNA and replication of the full-length RNA genome occur in the convoluted endoplasmic reticulum membranes. Finally, the full-length (+ sense) RNA genome binds to the nucleocapsid prior to full assembly of the virion, which eventually is released from the cell via exocytosis. Protein abbreviations: E, envelop; M, membrane; N, nucleocapsid; NSPS, non-structural; S, spike. The figure was adapted from [106] and created using Biorender.com.

The infectious dose of STEC and SARS-CoV-2 is comparable (Table 1) and indicates that small numbers of each pathogen are sufficient for the infection to proceed. In both cases, disease progression starts with the attachment of the pathogen to body organ cells; these are the intestinal enterocytes and the airway epithelium, respectively (Figure 4 and Figure 5). In the case of STEC, this attachment is facilitated by bacterial cell’s fimbria, intimin, and a translocated intimin receptor (Figure 4). Subsequently, the attached STEC secretes Shiga toxin (Stx), the pathogen’s main virulence factor, which binds to a receptor (globotriaosylceramide, Gb3, or globotetraosylceramide, Gb4) on the host cell. In contrast, the attachment of SARS-CoV-2 to the target host cell is more straightforward [106]. The S protein on the virus surface binds to the host cell’s ACE2 receptor (Figure 5). Subsequent processing of S protein by target cell proteases, transmembrane serine protease 2 (TMPRSS2) or cathepsin B, primes the S protein to mediate fusion between the viral envelope and cell membrane. It is expected that innate immune defenses act on the infection by both pathogens, and the virulence agent attempts to evade the immune response. Critically, for both STEC and SARS-CoV-2, excessive stimulation of the innate immune response can increase disease severity. As the disease progresses, infection of other organs may occur; these are the kidney and brain in case of STEC infection, and the heart, kidney, liver, brain, and blood vessels for SARS-CoV-2 infection.

The key factors in STEC pathogenesis are Stx1, Stx2, or both, and the products of the *LEE* operon. The *LEE* operon is composed of 5 regions; region 1–3 encode type III secretion system, region 4 encodes translocating proteins, and region 5 encodes intimin, which is essential for adhesion and attachment [22]. In STEC strains deficient in *LEE*, other factors contribute to adhesion such as the STEC autoagglutinating adhesin, Saa [107], autotransporter protein, Sab [108], *E. coli* immunoglobulin-binding proteins [109], and STEC adherence protein, ToxB [110]. All STEC strains can produce Stx as the main mechanism of disease whereas EHEC additionally possess the *LEE* operon.

The mechanism of STEC-associated diseases has been discussed in serval publications [22,111]; a summary of these mechanisms is illustrated in Figure 4. The infection begins when an STEC-contaminated food is ingested, the bacterium survives the acidity of the stomach and reaches the intestine, and pathogen cells colonize the mucosa of the lower gastrointestinal tract. The initial contact of STEC with the intestinal enterocytes occurs via fimbrial attachment, which helps the bacterium to interact with the enterocyte surface. STEC then expresses a virulence protein (translocated intimin receptor, Tir), which is translocated to the enterocyte membrane; Tir serves as an STEC attachment site. The STEC cell also expresses on its surface the protein intimin, which has affinity for Tir. The binding of intimin with Tir makes STEC become firmly attached to the enterocyte surface. This binding is followed by the accumulation of actin filaments, which is mediated by the effector Tir cytoskeleton-coupling protein, EspFu, leading to the creation of attaching/effacing (A/E) lesions. Subsequently, STEC secretes Stx that binds to the receptor Gb3 or Gb4; these receptors are found on the enterocyte surface. Stx is then internalized within a vesicle into the enterocyte via an endocytosis mechanism. The toxin is subsequently trafficked through the Golgi apparatus and endoplasmic reticulum, where the subunit A of the Stx separates and binds to 28s rRNA. Subunit A removes adenine residues from the 28s RNA molecules, leading to inhibition of the protein synthesis and consequently cell death.

Upon infection with STEC, the innate immune response is triggered to eliminate the pathogen, but evasion of such immunity could worsen the disease. Briefly, once the bacterial signals are recognized by host cells, an increased release of low-molecular-weight proteins, the cytokines, starts to mount [31,112]. Cytokines mediate cell-to-cell communication, initiate innate immunity, and then bind to specific cell membrane receptors to alter cellular gene expression [113]. Successful orchestration of these inflammatory responses could lead to pathogen elimination. However, excessive inflammatory responses could lead to harmful effects such as septic shock or organ failure. Nevertheless, STEC has developed means to evade the human immune response. During the colonization stage of STEC, the expression of effector genes encoded on the *LEE* operon leads to the accumulation of actin filaments (Figure 4), forming a raised pedestal and an increased intestinal barrier to permeability [114,115]. After translocation of effector proteins (e.g., Esp proteins or NleA-F) produced by STEC into the epithelial cells, the bacterium starts to induce low levels of the cytokines IL-6, IL-8, and IL-1α or suppress the nuclear factor-ΚB (NF-ΚB) pathway [116]. These events help in counteracting the host immune defenses at the stage of STEC colonization. At the HUS development stage, the acute inflammatory responses increase due to elevated production of IL-1, IL-6, TNF-α, and C-reactive proteins and T-cell activation [117,118]. The kidney is the primary target for Stx, and once these toxins enter the bloodstream, production of IL-6, IL-8, and IL-1β is induced in human proximal tubular cells [119]. In addition, researchers found that macrophages are recruited to the kidney after injection with Stx2 in a murine model characterized by renal damage [120]. These overall inflammatory responses contribute to kidney failure by Stx.

**Figure 6:**
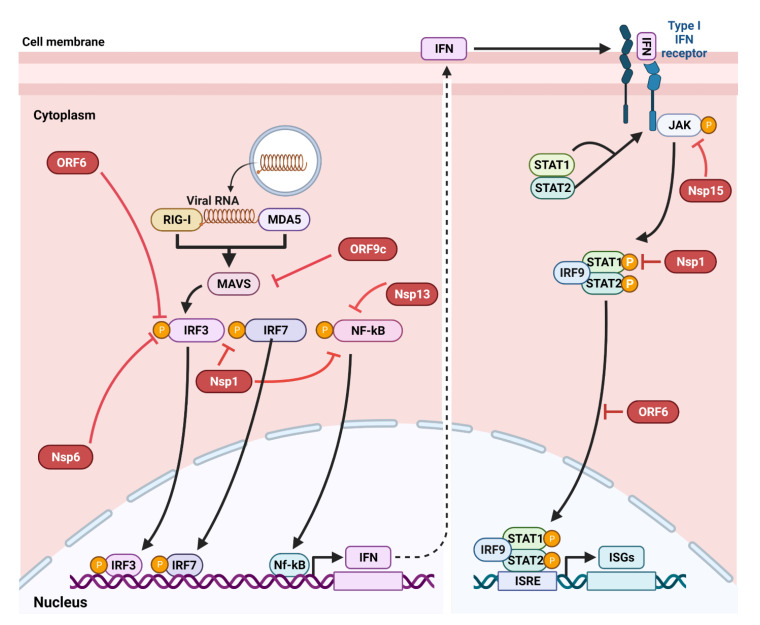
Evasion of interferon (IFN) pathways by SARS-CoV-2. The IFN responses start by sensing the viral RNA by pattern recognition receptors (PRRs, e.g., retinoic acid-inducible gene I (RIG-1)) and melanoma differentiation-associated protein 5 (MDA-5), which mediate signal transduction via the adaptor complex of the mitochondrial antiviral-signaling protein (MAVS). The PRR–adaptor interaction recruits kinases that phosphorylate interferon regulatory factor 3/7 (IRF3/7) and the nuclear factor-kB (NF-kB); these are transcriptional factors that enter the nucleus and transcribe IFNs. The IFNs act as a signal through Janus kinase 1 (JAK1) and the signal transducer and activator of transcription 1 and 2 (STAT1/2), resulting in the transcription of IFN-stimulated genes (ISGs), which exhibit antiviral effects. SARS-CoV-2 proteins encoded by open reading frame (ORF) 6 and 9c and several nonstructural proteins (Nsps) inhibit these pathways and render the virus resistant to IFN responses. The figure was adapted from [106] and created using Biorender.com.

In severe COVID-19 cases, following inhalation of SARS-CoV-2, the virus travels through the airways, where it binds to ACE2 receptor expressed on the airway epithelium and blood vessel endothelium. Upon entry of the virus inside these cells, the viral RNA undergoes a sophisticated process of translation and replication to generate new virions that are the infectious agents to neighbor cells (Figure 5). In parallel to the genomic RNA transcription and replication, the viral RNA is recognized by host pattern recognition receptors (PRRs; Figure 6) such as retinoic acid-inducible gene (RIG-1), melanoma-differentiation-associated protein (MDA-5), and toll-like receptors (TLR-7 and TRL-8) [21]. Subsequently, a cascade of signaling events leads to the expression of pro-inflammatory cytokines such as IL-8, IL-6, CCL-2, and CXCL9/10/11 [121,122,123]. The increased secretion of these cytokines during virus infection leads to the recruitment of immune cells, mainly macrophages and neutrophils. The excessive accumulation of cytokines and immune cells leads to what is known as a “cytokine storm”, which can promote tissue damage, compromise the endothelial junction, and thus cause vascular leakage [21,124,125]. This hyperinflammatory response leads to lung edema, limiting gas exchange in the lung and causing shortness of breath or irreversible respiratory failure [21]. In severe COVID-19 cases, patients experience increased proinflammatory cytokines in the bronchoalveolar lavage fluids, prolonged prothrombin time, and/or pneumonia, among other symptoms [122,123,126]. The excessive secretion of cytokines can also inflame multiple body organs (heart, kidney, small bowel, liver), leading to systemic multiorgan damage.

Similar to SARS-CoV-2 infections, the hyperinflammatory responses induced by Stx can cause body organ failures. Within this context, Stx released by STEC in the gastrointestinal tract is absorbed into the systemic circulation, where it binds to the vascular endothelial cells. This binding is followed by endothelial injury induced by a cascade of events that includes inhibition of protein synthesis, increased inflammatory and cytokine responses, and a triggered ribotoxic stress response. Eventually, such endothelial injury leads to the formation of microthrombi (i.e., vasculature thrombogenicity) and damage to target organs, mainly the kidneys and brain [28].

In line with STEC infection, evasion of the innate immune response is a key mechanism in SARS-CoV-2 pathogenesis. A crucial immune evasion process during SARS-CoV-2 infection is the ability to escape the primary innate interferon (IFN) pathways, which are potent in eliminating the virus infection (Figure 6). The IFN pathway starts with the recognition of the viral nucleic acid via pattern recognition receptors (PRRs), which perpetuate signaling events by interacting with adaptor proteins (e.g., mitochondrial antiviral-signaling proteins (MAVS)). Then the PRR-adaptor complex recruits kinases, which phosphorylate several transcriptional factors such as interferon regulatory factor 3/7 (IRF3/7) and NF-kB [21,32,127]. The phosphorylated transcriptional factors induce transcription of type I and III IFNs. The expressed type I and III IFNs then act as a signal through Janus kinase 1 and activators of transcription 1 and 2 (STAT1/2), which, in turn, induce the expression of IFN-stimulated genes (ISGs) [128]. However, SARS-CoV-2 possesses genes encoding inhibitory proteins (e.g., CoV-2 ORF9c, CoV-2 nsp13, CoV-2 nsp6, CoV-2 nsp1), which halt or inhibit the expression of IFN type I and III or even the ISG expression throughout this pathway as shown in Figure 6 [21]. These SARS-CoV-2 proteins are thought to have stronger inhibitory effects on IFN than those of SARS-CoV or MERS-CoV [32]. SARS-CoV-2 replicates more efficiently than SARS-CoV in ex vivo lung tissues, presumably due to the superior suppressive effects of SARS-CoV-2 proteins on IFN expression [129]. Since IFNs are important for early viral control in the host, more research is needed to decipher whether the IFNs’ suppression by SARS-CoV-2 correlates well with the lack of symptoms observed early in infection or with the rapid transmission of this virus.

## 5. Adaptive Immunity

The host immunity in response to SARS-CoV-2 infection is well documented, but evidence for such expression is not well characterized in the case of STEC infection. Neutralizing antibodies for Stx1 have been reported in the sera of STEC-infected patients, but the frequency of encountering these antibodies was low [130]. These researchers attributed their observation to the inadequate antigenicity of Stx1. On the contrary, anti-Stx2 neutralizing antibodies were detected in STEC-infected patients [131,132]. According to Ludwig et al. [133], the anti-Stx2 response following infection by Stx2-producing strain was 71% among 38 studied cases. Several attempts to produce monoclonal or polyclonal IgG against Stx1 and Stx2 were completed, and some of these antibodies are currently at the clinical trial stage [134]. However, there are many challenges in reaching the desirable anti-Stx neutralizing antibodies; these challenges include that STEC animal models (e.g., mice and pigs) do not develop HUS, as humans do, making it difficult to gauge the efficacy of the neutralizing antibodies, and the occurrence of STEC infections as outbreaks exacerbate the problem of enrolling volunteers in clinical trials.

Adaptive immunity plays a key role in eliminating SARS-CoV-2 infection. Three key players in SARS-CoV-2-specific immune responses in humans are the CD4^+^ T cells, CD8^+^ T cells, and antibodies. CD4^+^ T cells are detected in almost all individuals infected with SARS-CoV-2 and in higher abundance than CD8^+^ T cells [135,136]. The magnitude of SARS-CoV-2-induced CD4^+^ T cells’ response is correlated with the expression of the viral proteins. The S protein, M protein, and nucleocapsid are the main target for CD4^+^ T cells [135]. In response to SARS-CoV-2 infection, CD4^+^ T cells can be detected within 2–4 days of symptom onset, and the abundance of these cells are thought to have the strongest correlation with decreased COVID-19 severity [137]. COVID-19 patients with mild disease severity and rapid viral clearance were associated with rapid induction of CD4^+^ T cells and vice versa [138]. CD4^+^ T cells have multifaceted functions during SARS-CoV-2 infection. These include the ability of CD4^+^ T cells to differentiate into Th1 cells, which, in turn, have antiviral activities via the production of IFNγ [135,139]; T follicular helper (Tfh) cells, which help B cells in the development of neutralizing antibodies, and the long-term humoral immunity [140]; or CD4-CTL cells, which have direct cytotoxicity against several viral infections [141]. CD4^+^ T cells contribute to CD8^+^ T cell responses [142] and make cytokines such as IL-22, which participate in lung tissue repair [143]. CD8^+^ T cells are crucial for viral clearance because of their ability to kill infected cells and are associated with better COVID-19 outcomes [137]. CD8^+^ T cells exhibit high levels of cytotoxic effectors such as IFNγ, perforin, and granzyme B [136,137,144]. These effectors can provide antiviral activity during SARS-CoV-2 infection.

SARS-CoV-2-infected patients could develop seroconversion 5–15 days post-symptom onset, with the majority seroconverting by 10 days [137]. The S protein is the primary target for neutralizing antibodies [145], which can be produced from naive B cells during SARS-CoV-2 infection [146]. High antigen load is commonly known to induce high antibody titers, and this is true for SARS-CoV-2 infections [147].

The immunological mechanism against SARS-CoV-2 can be simplified as follows. Neutralizing antibodies stop the virus outside of cells whereas T cells stop the virus inside of cells. This is a successful complementary approach to mitigate SARS-CoV-2. However, it is worth mentioning that adaptive immunity takes time to develop, and the neutralizing antibodies cannot clear ongoing SARS-CoV-2 infection; instead, this task is led by the T cells [137,143]. This finding implies that neutralizing antibodies provide the protective immunity against SARS-CoV-2 infection spread inside the host, whereas clearing an ongoing infection is substantially provided by the T cell responses.

## 6. Emergence of STEC and SARS-CoV-2: Two Sides of One Story

It can be concluded from the previous discussion that SARS-CoV-2 and STEC emerged by comparable mechanisms, and infection progression by both agents has similarities, but there are also substantial differences (Table 1). Both agents have zoonotic potential (i.e., crossing from animal into humans), both evolved using gene acquisition and point mutations, and both developed host immune evasion mechanisms. Moreover, both agents have small infectious doses (≤100 PFU or CFU), and both require specific receptors to bind to the host cell and initiate a downstream signaling cascade leading to hijacking of the host cell’s physiology, which eventually leads to host cell death. Under severe infections, both agents result in organ failure. In contrast, the key differences stem from the fact that SARS-CoV-2 resulted in a pandemic within few months from the initial disease detection; however, STEC causes disease outbreaks that tend to be geographically localized, and it takes a long time to spread within a country or across continents. STEC is a foodborne infection while SARS-CoV-2 is a respiratory infection, but both agents affect the gastrointestinal tract, and both typically damage other body organs. STEC evolved mainly via gene acquisition (horizontal gene transfer and transduction) and gene deletions, whereas SARS-CoV-2 evolves mainly by genetic recombination and point mutations. Vaccines for SARS-CoV-2 were developed within a year of the pandemic beginning, whereas no commercial vaccine has been produced for STEC, although the pathogen’s associated infection was detected four decades ago. Lastly, both pathogens continue to evolve under immune pressure (SARS-CoV-2) or due to imposed environmental/animal host stress (STEC). Examining the evolutionary strategies employed by both pathogens may enable us to predict what is evolving now and what future emerging infectious agents could be.

SARS-CoV-2 and STEC are only two members of a long list of biological infectious agents that threatened humanity throughout history. With the proven genetic plasticity of these infectious agents and the intricate human interaction with nature, particularly wild animals, we must expect new emerging and re-emerging infectious pathogens to be provoked. Understanding the initial biological events leading to the emergence of these infectious agents could aid in avoiding future disease pandemics and outbreaks. Specifically, careful monitoring and perhaps regulation of the human/animal interface may be a key strategy for preventing future zoonotic transmission events.

The evolution of SARS-CoV-2 and STEC taught us that mutations will continue to accumulate, creating new emergent strains with changeable pathogenicity but with improved transmissibility. It seems that when these emerging strains become dominant and positively selected, their infectious doses tend to be small (Table 1). Additionally, emerging mutants tend to express resistance to disease prevention control measures (e.g., neutralizing antibodies for SARS-CoV-2), which alerts us to revisit current vaccination programs regularly. This effort may be achieved by encouraging efforts to constantly implement whole genome sequencing of infectious agents worldwide, particularly in low-resource countries, to trace the evolution and to keep up with the genetic make-up of infectious strains that are circulating. An alternative strategy to mitigate resistance to neutralizing antibodies is to adopt antiviral compounds that suppress the virus replication and could stop the infection inside the host cells. Examples of antivirals include Nirmatrelvir and Molnupiravir, which provided promising findings against several SARS-CoV-2 variants of concern. Moreover, continuing scientific investigations may alert us about infectious agents that mutate at the current moment.

## Figures and Tables

**Table 1:** Comparison of the characteristics of emergent Shiga toxin-producing *Escherichia coli* (STEC) and severe acute respiratory syndrome coronavirus 2 (SARS-CoV-2).

Criteria	STEC	SARS-CoV-2	Selected References
Agent	Gram-negative bacterium that carries its virulence genes on a prophage	Enveloped single-stranded positive-sense RNA virus	[1,2,20]
Host cells for initial entry	Intestinal enterocytes	Airway epithelium	[21,22]
Key virulence factor	Shiga toxin	Spike protein/viral and host proteases	[1,21,22,23,24]
Virulence factor receptor	Gb3 or Gb4	ACE2 receptor	
Infectious dose	~10–100 cells	~30–100 virions	[25,26,27]
Important disease culprit	Microthrombi in target organs	Cytokine storm and microthrombi in target organs	[28,29]
Target organs	Initially intestinal epithelium but kidney and brain are affected	Initially respiratory system but causesdamage in liver, kidney, brain, blood vessels, heart, and other organs	[28,30]
Host immune response	Innate immunity	Innate and adaptive immunity	[21,31,32]
Disease spread	Localized/regional outbreaks	Pandemic	[1,14,20]
Animal reservoir	Present	Present	
Zoonosis	Zoonotic	Zoonotic	
Transmission route	Mostly foodborne, with no person-to-person transmission	Respiratory droplets, contact with contaminated surfaces, aerosols	[20,33]
Response to non-vaccine therapeutics	Antibiotics can increase the severity of the disease; hence, it is not a recommended treatment	Some drugs exhibited antiviral activity ^a^	[34]
No. of illnesses/deaths	2,801,000/230 ^b^	543,733,163/6,329,375 ^c^	[35]
Serotypes/variants	>200 serotypes ^d^	>16 variants ^e^	[36]
Vaccine	Not available	Available	[37]
Evolution speed ^f^	Slow (years)	Fast (months–years)	[38,39,40,41]
Evolution mechanisms	Gene loss/genetic acquisition	Selection by mutation; genetic recombination	
Survival outside the host	weeks–months	Hours–several days	[20,42]

^a^ Data from the Food and Drug Administration (https://www.fda.gov/media/155054/download, accessed on 12 July 2022). ^b^ Data as of 2014. ^c^ Data as of 27 June 2022 (https://coronavirus.jhu.edu/map.html). ^d^ Approximately 50 non-O157 serotypes in addition to the O157 type were involved in human illnesses. ^e^ Data as of 25 May 2022 (https://www.ecdc.eropa.eu/en/covid-19/variants-concern). ^f^ The rapidity for new serotypes or variants to emerge.
